# Establishment of Hospital Emergency Incident Command System (HEICS) in Iranian Hospitals: A Necessity for Better Response to Disasters

**DOI:** 10.5812/ircmj.3371

**Published:** 2013-12-05

**Authors:** Mohammad Hossein Yarmohammadian, Golrokh Atighechian, Abbas Haghshenas, Lida Shams

**Affiliations:** 1 Health Management and Economic Research Center, Isfahan University of Medical Sciences, Isfahan, IR Iran; 2 Department of Health in Disasters and Emergencies, Tehran University of Medical Sciences, Tehran, IR Iran; 3 Department of Health Policy, Planning and Evaluation Curtin University, Curtin, Australia; 4 Department of Health Policy, Tehran University of Medical Sciences, Tehran, IR Iran

**Keywords:** Hospital Emergency Incident Command System, Hospital Management, Health Policy and Planning

Dear Editor,

Preparing and increasing efficiency in hospitals in disasters need to launch a common protocol named Hospital Emergency Incident Command System (HEICS). HEICS provide more coordination between hospitals and other institutions involved in emergency incidents by utilizing logical management structure, duty descriptions, creating clear reporting channels as well as developing a common and simple nomenclature system. Researches indicated that applying and establishing HEICS is necessity for Iranian hospitals and it can be helpful for use in both emergency and non-emergency situations, such as moving the facilities dispensing medications to hospital staff, or planning for a large hospital or community event. Developing training program on HEICS for hospital managers as well as establishing it seems necessary regarding our country’s condition. In this paper we point out establishing HEICS have to be in agenda for policy maker’s managers in Iranian health system and affiliated hospitals.

Critical incidents are sudden, unanticipated events or situations that require quick decisions. Experiences from all around the world show that confusion and chaos are the most common issues which are confronted in hospitals when there is a serious crisis (1). Clearly a timely and effective management system with accurate organization and proactive plans and activities could minimize negative consequences of such incidents and maximize efficiency of emergency services (2-4).

 Incident command system (ICS) is currently the most common system of incident management in the world, gaining more recognition due to its successful outcomes (2, 4). In ICS, the incident commander, is responsible for directing other peoples to be well organized and involved. ICS is founded on some basic principles, which ensure using resources effectively, and on the other hand, reduce disturbances in decision making and doing proper operations, by responsible organizations (5). In other words, successful response to crisis requires coordination among all hospital wards and units as well as collaboration with pre-hospital emergency forces (such as EMS, Red Crescent agency), police, firefighting in order to make sure communication lines are established. Although certain level of flexibility needed to respond to variety of crisis (6).

 Instead of ICS, preparing and increasing efficiency in hospitals in such circumstances, a common protocol named Hospital Emergency Incident Command System (HEICS) has been proposed which is extracted from basis ICS (2). HEICS was created in the late 1980s as an important foundation for the 5815 registered hospitals in the United States in their efforts to prepare for and respond to various types of disasters (7). In fact, HEICS is an incident management system composed of some specific roles in the form of an organizational table. Each of these roles has a specific mission during crisis situation, and there is a list of individual job description which guide appointed person in the crisis situation carefully. Actually HEICS allows providing as much responsibilities as required at any time, and it means more effectiveness and lower cost. Thus HEICS has capability of adapting with different kinds of incidents and crisis in any scale (8-10). HEICS provide more coordination between hospitals and other institutions involved in emergency incidents by utilizing logical management structure, duty descriptions, creating clear reporting channels as well as developing a common and simple nomenclature system (2, 4, 11).

HEICS provides a commanding system which doesn’t rely on specific people, but it is flexible (6). Significant and effective features in HEICS, especially in its third version (1998) have made it one of the most common and most comprehensive hospital management systems. In developing the fourth edition of HEICS, the value and importance of using an incident management system to assist as well with daily operations, preplanned events, and non-emergency situations became apparent. Thus, the HEICS was created as a system for use in both emergency and non-emergency situations, such as moving the facility, dispensing medications to hospital staff, or planning for a large hospital or community event (7). Developing training program on HEICS for hospital managers as well as establishing it seems necessary regarding our country’s condition.

In spite of the necessity of establishing such a system, many developing countries still do not have to establish this system. According Yarmohammadian and his colleagues, barriers for HEICS establishment in Iranian hospitals are classified into two categories: internal and external. Internal barriers are related to health care sector and external barriers are referred to out of health care sector. Some of internal barriers include high cost implementation, lack of motivation in the hospital managers and staff, lack of common language, lack of competitive atmosphere for progress and excellence, involvement of administrative managers in daily activities, lack of empowerment, lack of feeling the need for crisis management, and lack of knowledge in managers. External barriers include lack of authorities support and non-commitment of managers, lack of qualified managers, absence of statutory requirements, too many decision maker authorities, lack of administrative culture for crisis management, poor communication and coordination in crisis team, constant change in regulations, and lack of emergency incident command system in the country (12).

According to these barriers some solutions are: using other people’s experience and preparation of training packages related to unexpected disasters, support of department of ministry of health and considering extra and specific budget for it, concept building in different dimensions, creating interest in personnel, appointing qualified managers, training and informing managers in all levels in order to create shared language among them and culture-making, designing instructions and legal bylaws for hospitals and legal requirements, elimination of daily and limited concerns of managers and removing complex administrative processes, inclusion of emergency incident management in managers’ job description and regarding it in evaluation of hospital managers, preparation of indexes and standards related to implementation of circular by managers and selection of managers, job description and monitoring of managers, establishment of regular committee for managers so that they can have planning and implementation of this system in macro level and creating a single organization in the whole community (12-14).

It is concluded that, since this system is one of the necessary needs of hospitals, it is recommended that such barriers should be eliminated and responsible entities including Ministry of Health and Medical Education (MoHME) and affiliated hospitals provide codified and ordered plans for establishment of this system. MoHME through approving and notifying rules and regulations can be established such valuable and proactive system for affiliated hospitals as well as private ones.
